# Cholesterol content regulates silica-induced lysosomal membrane permeability

**DOI:** 10.3389/ftox.2023.1112822

**Published:** 2023-02-13

**Authors:** Matthew J. Sydor, Rebekah L. Kendall, Andrij Holian

**Affiliations:** ^1^ Center for Environmental Health Sciences, Department of Biomedical and Pharmaceutical Sciences, University of Montana, Missoula, MT, United States; ^2^ Center for Biomolecular Structure and Dynamics, University of Montana, Missoula, MT, United States

**Keywords:** silica, macrophage, lysosome, inflammation, membrane

## Abstract

Inhalation of crystalline silica has been well documented to cause pulmonary inflammation and lung disease such as silicosis. Respirable silica particles deposit in the lungs and are phagocytosed by alveolar macrophages. Subsequently, phagocytosed silica remains undegraded within lysosomes causing lysosomal damage known as phagolysosomal membrane permeability (LMP). LMP can trigger the assembly of the NLRP3 inflammasome resulting in release of inflammatory cytokines that contribute to disease. In order to better understand the mechanisms of LMP this study used murine bone marrow derived macrophages (BMdM) as a cellular model to investigate the mechanism of silica-induced LMP. Reduction of lysosomal cholesterol in bone marrow derived macrophages with 18:1 phosphatidylglycerol (DOPG) liposome treatment increased silica-induced LMP and IL-1*β* release. Conversely, increasing lysosomal and cellular cholesterol with U18666A reduced IL-1*β* release. Co-treatment of bone marrow derived macrophages with 18:1 phosphatidylglycerol and U18666A resulted in a significant reduction of the effects of U18666A on lysosomal cholesterol. Phosphatidylcholine 100-nm liposome model systems were used to examine the effects of silica particles on lipid membrane order. Time-resolved fluorescence anisotropy of the membrane probe, Di-4-ANEPPDHQ, was used to determine changes to membrane order. Silica increased lipid order that was attenuated by inclusion of cholesterol in the phosphatidylcholine liposomes. These results demonstrate that increased cholesterol can attenuate silica-induced membrane changes in liposomes and cell models, while decreasing cholesterol exacerbates silica-induced membrane changes. Selective manipulation of lysosomal cholesterol may be a way of attenuating lysosomal disruption and preventing silica-induced chronic inflammatory disease progression.

## 1 Introduction

Inhalation of crystalline silica particles has been extensively documented in the development of the fibrotic lung disease, silicosis (Davis, 1986; Cullinan et al., 2017; Hoy and Chambers, 2020). Inhaled, silica particles are deposited in various locations in the lungs, dependent on their size. Some of the smaller particles (less than 2 µm in size) can deposit in the alveolar space, where they will be phagocytosed by alveolar macrophages resulting in inflammation (Hamilton et al., 2006; Hornung et al., 2008; Darquenne, 2012). Phagocytosed crystalline silica has been reported to activate the NLRP3 inflammasome in alveolar macrophages (Hornung et al., 2008). This activation of the NLRP3 inflammasome induces caspase-1 activity, which cleaves the inflammatory cytokines pro-IL-1*β* and pro-IL-18 to their active forms for secretion (Dinarello, 1998; Hornung et al., 2008).

Current research is providing more details on the steps leading to silica-induced NLRP3 inflammasome activation. Phagolysosomal membrane permeability (LMP) and the release of degradative lysosomal enzymes such as cathepsins is the proposed mechanism by which particles can trigger NLRP3 inflammasome assembly (Jessop et al., 2017; Jia et al., 2019). When silica particles are present in the phagolysosome, they are not degraded and can cause a destabilization of the lysosome. Details of how silica causes LMP remain unclear. However, there are reports suggesting that silica can interact preferentially with phospholipid components of biological membranes and cause changes to the lipid order increasing permeability of those membranes (Kettiger et al., 2016; Wang et al., 2019; Sydor et al., 2021; Pavan et al., 2022). Key surface groups called nearly free silanols (NFS) have been described and are present on the fractured surface of crystalline silica and other types of quartz materials (Brinker et al., 2020; Pavan et al., 2020). The presence of NFS on particles has been associated with increased membrane disruption and toxicity when compared to particles with fewer or no NFS (Pavan et al., 2020; Pavan et al., 2022).

Because inhalation of crystalline silica remains an occupational hazard for many individuals, better understanding of the mechanism of silica-induced LMP should help develop potential therapeutics for silica-induced inflammation. With LMP being a critical step in this inflammatory pathway, modulation of the lysosome lipid composition may help to better understand and eventually prevent chronic disease. Increasing lysosomal cholesterol with the compound U18666A has been implicated in reducing levels of LMP (Appelqvist et al., 2011; Reiners et al., 2011; Appelqvist et al., 2012). Conversely, cells can be treated to reduce lysosomal cholesterol by increasing cholesterol trafficking. Treatment of cells with liposomes composed of phosphatidylglycerol (PG), a precursor to LBPA, has been demonstrated to increase LBPA levels in cells (McCauliff et al., 2019). Treatments of both LBPA and PG have been shown to reduce intracellular cholesterol accumulation in a Nieman-pick disease model (McCauliff et al., 2019; Ilnytska et al., 2021a; Ilnytska et al., 2021b) as LBPA has been reported to have interact with the NPC2 protein and increase its cholesterol trafficking function (McCauliff et al., 2019).

This current work examined how modulating lysosomal cholesterol content impacted crystalline silica-induced membrane disruption and LMP. While previous studies have demonstrated the regulation of LMP by cholesterol, we have examined this effect in conjunction with a relevant particle treatment. We hypothesized that a lack or reduction of cholesterol in membranes would lead to increased silica-induced membrane disruption and LMP. In these studies bone marrow derived macrophages (BMdM) were used as an *in vitro* macrophage model to study how manipulating lysosomal cholesterol affected LMP, cytotoxicity, and IL-1*β* release. BMdM were treated with liposomes composed of 18:1 phosphatidylglycerol (DOPG) prior to silica exposure to investigate how a reduction in lysosomal cholesterol impacted LMP. Finally, liposomes with a diameter of 100-nm were used as model membranes to study silica-induced changes to membrane order and how the presence of cholesterol affected these changes.

## 2 Materials and methods

### 2.1 Materials and reagents

The phospholipids 1,2-dioleoyl-sn-glycero-3-phospho-(1′-rac-glycerol) (18:1 Δ9-cis) (DOPG) (Item No. 840475) and 1,2-dioleoyl-sn-glycero-3-phosphocholine 18:1 (Δ9-Cis) (DOPC) (Item No. 850375) were purchased in chloroform from Avanti Polar Lipids, inc. (Alabaster, AL). Cholesterol powder (Item No. 700000) was also bought from Avanti Polar Lipids, inc. (Alabaster, AL). The fluorescence probe used for anisotropy measurements was Di-4-ANEPPDHQ, which was purchased from ThermoFisher (Waltham, MA). U18666a (Item No. 10009085) was obtained from Cayman Chemical (Ann Arbor, MI). The fluorescent substrate for n-acetyl-*β*-glucosaminidase was 4-Methylumbelliferyl-2-acetamido-2-deoxy-β-D-Glucopyranoside (Item No. 26953), and it was purchased from Cayman Chemical (Ann Arbor, MI). Filipin complex (Item No. F9765), digitonin (Item No. 300410), and Pefabloc SC (Item No. 11429868001) were from Sigma Aldrich (St. Louis, MO). For staining lysosomes, LysoView 633 (Item No. 70058) was purchased from Biotium, Inc. (Fremont, CA). The Cellview cell culture dishes (Item No. 627871) were from Greiner Bio-One North America, Inc. (Monroe, NC). Lactate dehydrogenase (LDH) in the cellular supernatant was measured with a CytoTox 96 Non-radioactive Cytotoxicity Assay (Item No. G1780) Promega (Madison, WI). Crystalline silica (Min-u-sil-5) was obtained from the Pennsylvania Glass Sand Corporation (Pittsburgh, PA). Min-u-sil-5 has a circle equivalent average diameter of 1.3 µm and has been further characterized in our previous work (Pavan et al., 2022).

### 2.2 Mice

C57BL/6J mice (Jackson Laboratories) were housed in the University of Montana’s specific-pathogen-free Laboratory Animal Resources facility with controlled environmental conditions and a 12 h light/dark cycle. Mice were euthanized by overdose of sodium pentobarbital (Euthasol™) prior to collection of legs for bone marrow. The animal procedures used in this study were approved by the Institutional Animal Care and Use Committee (IACUC) at the University of Montana.

### 2.3 Isolation and culture of bone marrow derived macrophages

C57Bl/6 mice were sacrificed, and the hind legs removed for bone marrow isolation. Bone marrow cells were collected by flushing the tibia and femur bones with complete media (RPMI, 10% FBS, 1% Penicillin/Streptomycin, 1% Sodium Pyruvate, 0.1% β-mercaptoethanol) with a 1/2” long 27-gauge needle. Cells were incubated overnight (37°C; 3.0 × 10^7^ cells/T75 flask) to eliminate stromal cells by adherence. The following day, non-adherent cells were transferred to new T75 flasks (1.5 × 10^7^ seeding density) with macrophage colony stimulating growth factor (M-CSF) (20 ng/mL, R&D Systems). Cells were maintained with additional M-CSF (10 ng/mL) every 3–4 days. BMdM were collected by trypsin and gentle scraping and used in all described experiments after day 9.

### 2.4 Cytotoxicity and cytokine analysis

BMdM were primed with 20 ng/mL lipopolysaccharide (Sigma) for NLRP3 inflammasome activation and treated with 100-nm DOPG liposomes (50 μM) for 1 h before silica (50 μg/mL) application. BMdM were incubated (37°C; 5% CO_2_) for 24 h in 96 well plate (100,000 cells/well) in complete RPMI 1640 media (10% fetal bovine serum, 1% Penicillin/Streptomycin). Cell death was measured in supernatants by Promega CytoTox 96 Non-Radioactive LDH assay. IL-1β release was quantified in supernatants by R&D Systems IL-1β ELISA kit.

### 2.5 Lysosomal membrane permeabilization assay

The measurement of lysosomal membrane permeability was adapted from methods that were previously described (Aits et al., 2015; Jessop et al., 2017). BMdM were plated at an amount of 1 × 10^5^ cells per well and were treated with 50 μg/mL silica and/or 50 µM DOPG liposomes. After 24 h, the supernatant was removed for use in an LDH assay. The cells were then washed twice with PBS before 100 µL of cytosolic extraction buffer was added. The composition of the cytosol extraction buffer was 250 mM sucrose, 20 mM HEPES, 10 mM KCl, 1.5 mM MgCl_2_, 1 mM EDTA, 1 mM EGTA, 0.5 mM pefabloc SC, and 15 μg/mL digitonin. A digitonin titration was performed in advance to select the optimal working concentration (15 μg/mL) of digitonin needed to fully permeabilize the plasma membrane but have minimal effect on lysosomes. The plate was then placed on ice with rocking for 15 min. After 15 min, the extracted cytosol was removed from the cells and placed in a new 96 well plate on ice. The activity of n-acetyl-*β*-glucosaminidase (NAG) was measured by adding 30 µL of extracted cytosol to 100 µL of NAG reaction buffer (0.2 M sodium citrate pH 4.5, 300 μg/mL 4-Methylumbelliferyl-2-acetamido-2-deoxy-β-D-Glucopyranoside). Cleavage of the fluorescent NAG substrate (4-Methylumbelliferyl-2-acetamido-2-deoxy-β-D-Glucopyranoside) was measured for 20 min with 45 s intervals at 30°C in a Molecular Devices (San Jose, CA) SpectraMax M4 microplate reader. The cleaved substrate was excited at 356-nm, and the fluorescence emissions were detected at 444-nm. Measured NAG activity was normalized by dividing NAG activity from the working concentration of digitonin (15 μg/mL) by the activity from the total lysis concentration of digitonin 200 μg/mL. This was done for each condition.

### 2.6 Cholesterol efflux

BMdM were added to a 96-well plate (100,000 cells per well) with TopFluor cholesterol (5 μg/mL) in serum-free media for 24 h (37°C), washed, and incubated with U18666A for 24 h or DOPG for 1 h in complete media (RPMI 1640, 10% FBS), in a similar fashion to that previously described (Liu et al., 2014). Supernatants were transferred to separate wells in black 96 well plate and TopFluor fluorescence measured by a Molecular Devices (San Jose, CA) SpectraMax M4 microplate reader. Efflux of treated BMdM was compared to control efflux. TopFluor was excited at 488-nm and detected at 525-nm.

### 2.7 Fluorescence imaging

All fluorescence images were collected on a Zeiss 880 LSM (Carl Zeiss Microscopy, LLC., White Plaines, NY). The objective used to collect the images was a ×63 Plan Apo 1.4 NA oil immersion objective. A confocal aperture of 1 AU was used for all image acquisition. For imaging of cholesterol and lysosomes, a procedure similar to that of (Kummer et al., 2022) was used. BMdM were seeded the prior day at 1.5 × 10^5^ cells per well in a 4-well Greiner Cellview cell culture dish. The next day, BMdM were stained with LysoView 633 for 1 h at a concentration of 1x in complete media per the manufacturer’s instructions. The cells were then washed twice with PBS before being fixed for 10 min at room temperature in 4% paraformaldehyde. Again, the cells were washed twice with PBS before addition of filipin staining solution in complete media. Filipin staining solution was made by diluting a 2.5 mg/mL stock of Filipin in DMSO 1:2 with 2% BSA/PBS. BMdM were then incubated at 37°C in the dark for 2 hours. The staining solution was then removed and replaced with phenol-red free complete media. Filipin was excited with a 405-nm laser, and LysoView 633 was excited with a 633-nm laser. The laser power and detector gain were held contestant across all conditions. At least six images of each condition were acquired per experiment. Experiments were conducted over three separate days. The laser power and detector gain were held constant across all conditions. Fluorescence overlays were generated with Zeiss Zen 2.3 software.

### 2.8 Liposome preparation and time-resolved fluorescence anisotropy

All lipids were dissolved in chloroform and allowed to dry under a stream of nitrogen gas for approximately 1 h. The dried lipids were rehydrated with Dulbecco’s PBS or tris buffered saline (TBS) (150 mM NaCl, 50 mM tris). The lipids were then sonicated in a water bath for 10 min prior to extrusion through an Avanti mini extruder set with a 100-nm pore size filter. DOPG liposomes in PBS were used to treat cells, while DOPC liposomes in TBS were incubated with silica particles at 37°C with tumbling for 2 h. Lipid order in 100-nm DOPC liposomes was assessed by time-resolved fluorescence anisotropy measurements of the lipophilic probe, Di-4-ANEPPDHQ. The fluorescence probe, Di-4-ANEPPDHQ has been previously demonstrated to be sensitive to changes in the lipid environment surrounding the probe (Owen et al., 2011; Steele et al., 2019; Sydor et al., 2020; Sydor et al., 2021; Pavan et al., 2022). Di-4-ANEPPDHQ was dissolved in spectral grade dimethyl sulfoxide (DMSO) and used at a working concentration of 400 nM with the final DMSO concentration being <1%. Di-4-ANEPPDHQ was added 15 min before data collection and was allowed to incubate with the liposome sample at room temperature, as previously described (Steele et al., 2019). An in-house built fluorimeter with parts from Quantum Northwest (Liberty Lake, WA) was used to acquire time-resolved anisotropy measurements as previously reported (Minazzo et al., 2009). A PicoQuant 470-nm pulsed diode laser (LDH-P-C-470) was used to excite the Di-4-ANEPPDHQ. Fluorescence emissions were collected with a Chorma 500-nm longpass filter and an Edinburgh Instruments photomultiplier tube (H10720-01). All data was collected from a sample chamber at 20°C ± 0.02°C. The anisotropy decay curves were fit using the PicoQuant FluoFit software (V4.6.6). The range of motion of Di-4-ANEPPDHQ in the lipid membrane was presented as a wobble-in-a-cone-angle. This was calculated from equation #1 and has been previously used to describe lipid membrane order (Steele et al., 2019; Sydor et al., 2020; Pavan et al., 2022).

### 2.9 Statistical analysis

Statistical significance was defined as *p* < 0.05. An unpaired-student’s t-test was used to compare the statistical difference of two means. For the comparison of more than two means, one or two-way ANOVA were used. A Dunnett post-test was used to compare multiple means back to the control. For comparisons between all groups, a Tukey HSD or Sidak-Holmes post-test was used. A minimum of three experimental replicates were performed for each data set. All statistical tests and graphs were generated with Prism 9.0 by GraphPad Software (San Diego, CA).

## 3 Results

### 3.1 Silica-induced IL-1β release from BMdM was decreased by U18666A and enhanced by DOPG treatment

**FIGURE 1:**
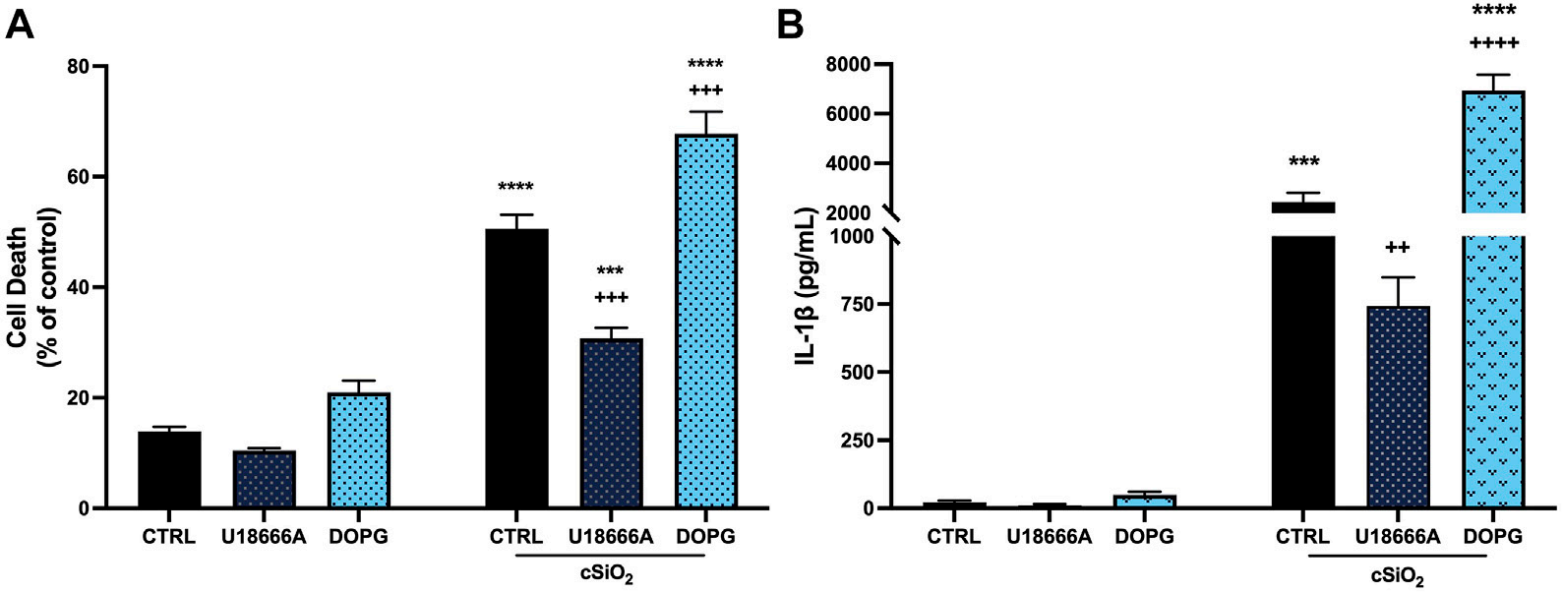
DOPG increased silica cytotoxicity and IL-1β release in BMdM with U18666A producing an opposite effect. LPS-primed BMdM were treated with and without U18666A (10 μg/mL) or DOPG (50 μM) for 1 h prior to silica (50 μg/mL) application in complete media. After 24 h, **(A)** cell death was assessed as LDH release in supernatants and **(B)** IL-1β release was measured by ELISA. Data expressed as *mean* ± *SEM*, *n* = 4 Analyzed by two-way ANOVA with Tukey's multiple comparisons test. *p < 0.05, **p < 0.01, and ****p < 0.0001 compared to control untreated cells. ^++^ *p* < 0.01 and +++ *p* < 0.001 compared to silica-exposed cells.

It has previously been demonstrated that crystalline silica promotes increased IL-1β release and cell death in BMdM (Hornung et al., 2008). Because NLRP3 inflammasome activation requires a priming as well as an activating signal, BMdM were primed with LPS (20 ng/mL) immediately prior to incubation with silica (50 μg/mL) for 24 h. Silica caused a significant increase in cell death measured by LDH release (Figure 1A) compared to untreated BMdM. This increase in cytotoxicity was accompanied by a significant increase in IL-1β release following silica exposure (Figure 1B).

The protective effect of cholesterol in lysosomal membranes was confirmed by addition of U18666A (10 μg/mL) to BMdM for 1 h prior to silica for 24 h. U18666A interacts with the NPC1 cholesterol transporter by binding the sterol-sensing domain and causes an increase in lysosomal cholesterol (Liscum and Faust, 1989; Lu et al., 2015; Chung et al., 2018). U18666A significantly reduced silica-induced cell death (Figure 1A) and IL-1β release compared to silica treated control BMdM (Figure 1B).

In contrast to U18666A, DOPG is reported to increase cholesterol trafficking out of the lysosome, thereby reducing lysosomal cholesterol (Ilnytska et al., 2021b). Here 100-nm liposomes comprised of DOPG (50 μM) were applied to BMdM for 1 h before 24 h silica treatment. While DOPG alone did not impact cell death compared to control BMdM, DOPG significantly increased cell death compared to silica-only treated BMdM (Figure 1A). Additionally, DOPG treatment significantly increased IL-1β release compared to silica-only BMdM (Figure 1B). These results demonstrate that U18666A treatment reduces silica-induced cell death and IL-1β release while DOPG treatment increases silica-induced cell death and IL-1β release in BMdM.

### 3.2 DOPG increased cholesterol efflux from BMdM

**FIGURE 2:**
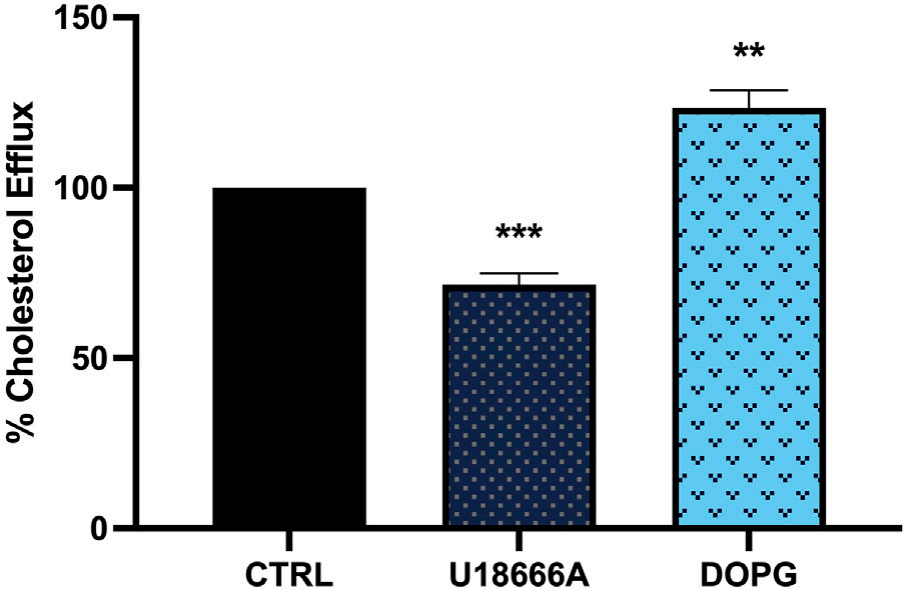
DOPG increased cholesterol efflux from BMdM with U18666A decreasing efflux. BMdM were loaded with TopFluor cholesterol (5 μg/mL) in serum free RPMI 1640 media. After 24 h, cells were washed and treated with U18666A (10 μg/mL) for 24 h or DOPG for 1 h in complete media. TopFluor fluorescence intensity measured in supernatants after 24 h. Data expressed as *mean* ± *SEM*, *n* = 4. Analyzed by one-way ANOVA with Dunnett’s multiple comparisons test. **p < 0.01 and ***p < 0.001 compared to untreated control cells.

DOPG and its derivative, lysobisphosphatidic acid (LBPA) are reported to increase cholesterol trafficking from lysosomes (McCauliff et al., 2019; Ilnytska et al., 2021a; Ilnytska et al., 2021b). Lysosomal cholesterol trafficking patterns impact cellular cholesterol efflux from macrophages, therefore the impact of U18666A and DOPG on cholesterol efflux was confirmed. BMdM were loaded with fluorescently labeled TopFluor cholesterol in serum-free conditions for 24 h. After washing, cells were treated with U18666A (10 μg/mL) for 24 h or DOPG (50 μM) for 1 h in complete RPMI 1640 media (10% FBS). Fluorescence intensity was measured in supernatants after 24 h and compared to untreated BMdM to assess cholesterol efflux. DOPG treatment significantly increased cholesterol efflux compared to untreated control BMdM (Figure 2). In contrast, U18666A treated BMdM demonstrated a significant decrease in cholesterol efflux (Figure 2). These results demonstrate that DOPG promotes cholesterol efflux from BMdM while U18666A prevents cholesterol efflux.

### 3.3 DOPG reduced U18666A-induced accumulation of lysosomal cholesterol in BMdM

**FIGURE 3:**
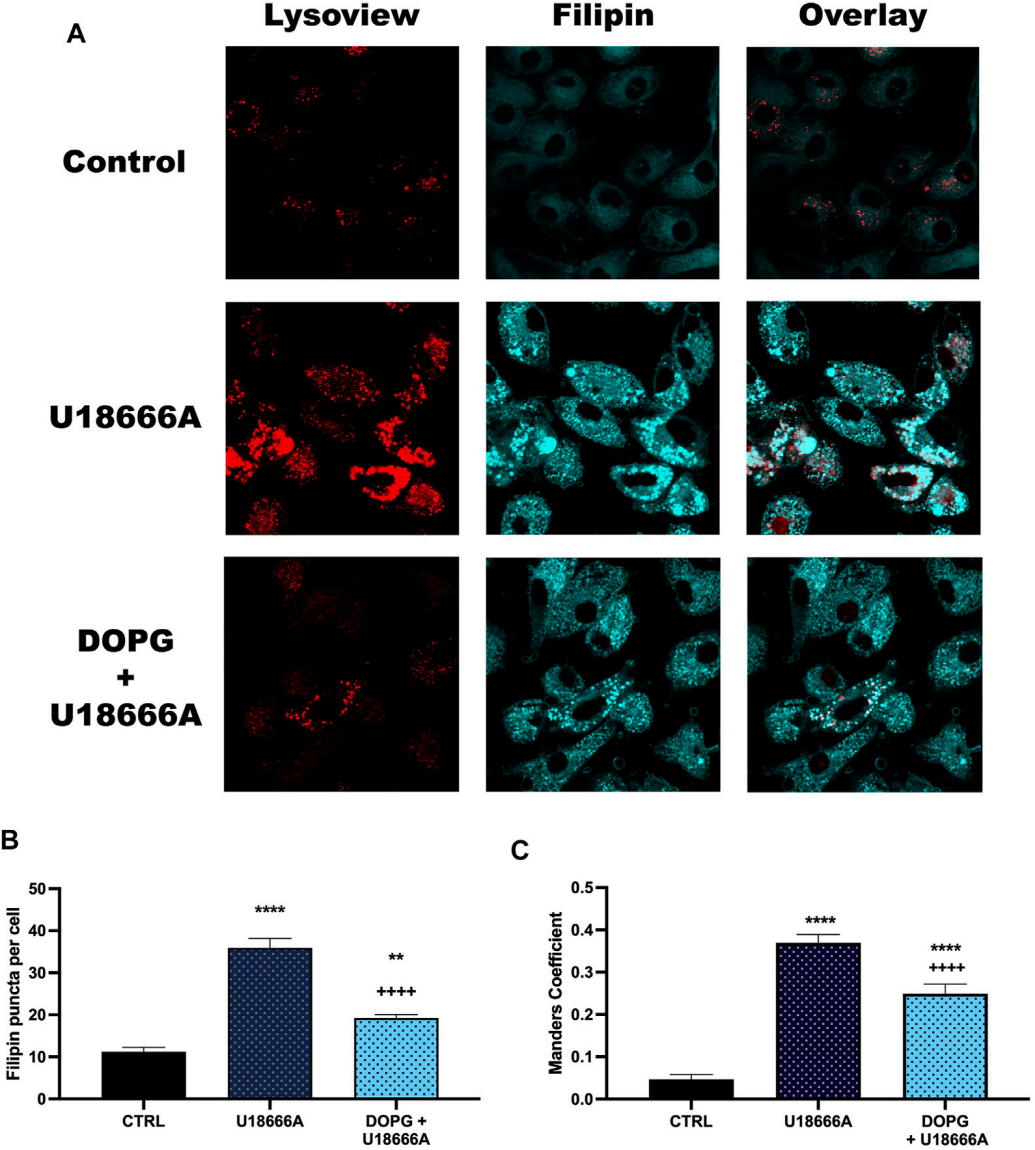
DOPG reduced U18666A filipin puncta. BMdM were treated with U18666A or DOPG + U18666A for 24 h. Cells were stained with Lysoview prior to fixation and filipin staining. **(A)** Representative images of Lysoview and filipin stained BMdM. **(B)** Filipin puncta per cell quantified by ImageJ analysis. **(C)** Manders colocalization analysis of Filipin staining with Lysoview staining. Data expressed as *mean* ± *SEM*, *n* = 3 independent experiments. Analyzed by one-way ANOVA with Tukey's multiple comparisons test. **p < 0.01 and ****p < 0.0001 compared to control untreated cells. ^++++^ *p* < 0.0001 compared to U18666A treated cells.

To demonstrate that the increased DOPG-induced cholesterol efflux corresponded to decreased lysosomal cholesterol upon DOPG treatment, free cholesterol within BMdM was measured by filipin staining. Filipin is a fluorescent antibiotic that binds unesterified (free) cholesterol in fixed cells (Maxfield and Wustner, 2012). Previously, it has been demonstrated that U18666A treatment causes an accumulation of filipin-stained free cholesterol within lysosomes (Reiners et al., 2011; Meyer et al., 2021). Here, BMdM were incubated with U18666A to demonstrate free cholesterol accumulation or DOPG + U18666A for 24 h to assess whether DOPG could reverse free cholesterol accumulation induced by U18666A. Treated BMdM were stained with Lysoview 633 prior to fixation and filipin staining. U18666A caused an observable increase in distinct puncta with Lysoview 633 and filipin staining compared to control BMdM indicating an accumulation of cholesterol within the lysosomes (Figure 3A). DOPG + U18666A-treated BMdM showed a similar colocalization of Lysoview 633 and Filipin as cholesterol is sequestered within the lysosomes (Figure 3A). Quantification of filipin stained puncta with ImageJ showed that U18666A significantly increased filipin puncta compared to untreated BMdM (Figure 3B). DOPG + U18666A treated cells also showed a significant increase in filipin puncta compared to untreated BMdM; however, they were significantly reduced in number compared to U18666A only treated cells (Figure 3B). DOPG alone produced no demonstratable change in filipin stained puncta, likely due to the considerably low number of observable puncta in control cells (data not shown). An analysis of the Manders Overlap Coefficient (using ImageJ JACoP) showed a significant increase in the amount of Lysoview 633 staining overlapping with filipin staining (Figure 3C) upon U18666A treatment, which was then significantly reduced by DOPG treatment.

### 3.4 DOPG decreased lysosomal cholesterol but not plasma membrane cholesterol in BMdM

DOPG is proposed to reduce lysosomal cholesterol by promoting increased trafficking of cholesterol through the NPC2 protein (McCauliff et al., 2019; Ilnytska et al., 2021a; Ilnytska et al., 2021b). In order to assess that lysosomal cholesterol was lowered, digitonin treatment was used. Digitonin is a cell membrane permeabilizer that specifically interacts with membrane cholesterol (Nishikawa et al., 1984). Therefore, since a change in membrane cholesterol would affect the efficacy of digitonin to permeabilize membranes, we assessed the impact of DOPG treatment on a digitonin titration curve compared to untreated BMdM. Increasing cholesterol content in the membrane corresponds with increased susceptibility to digitonin-induced lysis (Sudji et al., 2015). Detection of lysosomal enzymes in the extracted cytosol can then be used to assess lysosomal membrane integrity (Aits et al., 2015; Jessop et al., 2017).

**FIGURE 4:**
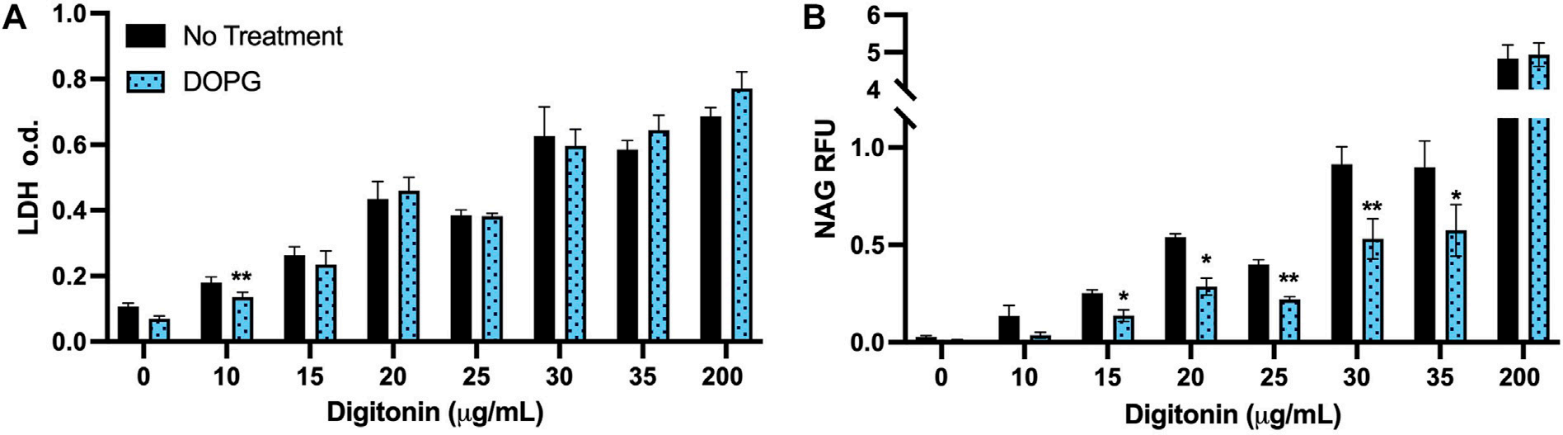
DOPG decreases lysosomal susceptibility to digitonin extraction. BMdM were titrated with digitonin (0–200 μg/mL) for 15 min on ice. **(A)** LDH optical density and **(B)** NAG relative fluorescence were measured in cytosolic extracts. Data expressed as *mean* ± *SEM*, *n* = 3. Analyzed by paired *t*-test, *p < 0.05 and **p < 0.01, between untreated and DOPG treated BMdM at respective digitonin concentration.

Digitonin was applied (0–200 μg/mL) for 15 min on ice, then cytosolic extracts were measured for the cytosolic enzyme lactate dehydrogenase (LDH) and the lysosomal enzyme n-acetyl-*β*-glucosaminidase (NAG). The highest dose of digitonin (200 μg/mL) was selected because it was much higher than the rest of the range and will permeabilize both the plasma membrane and lysosomal membranes in all conditions. DOPG treatment did not change LDH measurements compared to untreated BMdM with the exception of digitonin at 10 μg/mL (Figure 4A). In contrast, NAG values were significantly decreased compared to control BMdM at multiple digitonin concentrations (15–35 μg/mL) (Figure 4B).

The lack of demonstratable difference between LDH values for BMdM with and without DOPG treatment (Figure 4A) suggests that the digitonin extraction is extracting the cytosol to a comparable degree between the two groups due to their similar plasma membrane cholesterol levels. The significant decrease in measured NAG fluorescent intensity between untreated and DOPG treated BMdM points to a significant difference in lysosomal cholesterol content between the two groups, as the lower lysosomal cholesterol of the DOPG treated BMdM would make it less susceptible to digitonin extraction. Thus, these results demonstrate that DOPG treatment had no significant impact on plasma membrane cholesterol but significantly lowered the lysosomal cholesterol content.

### 3.5 Silica-induced LMP in BMdM was enhanced by DOPG treatment

**FIGURE 5:**
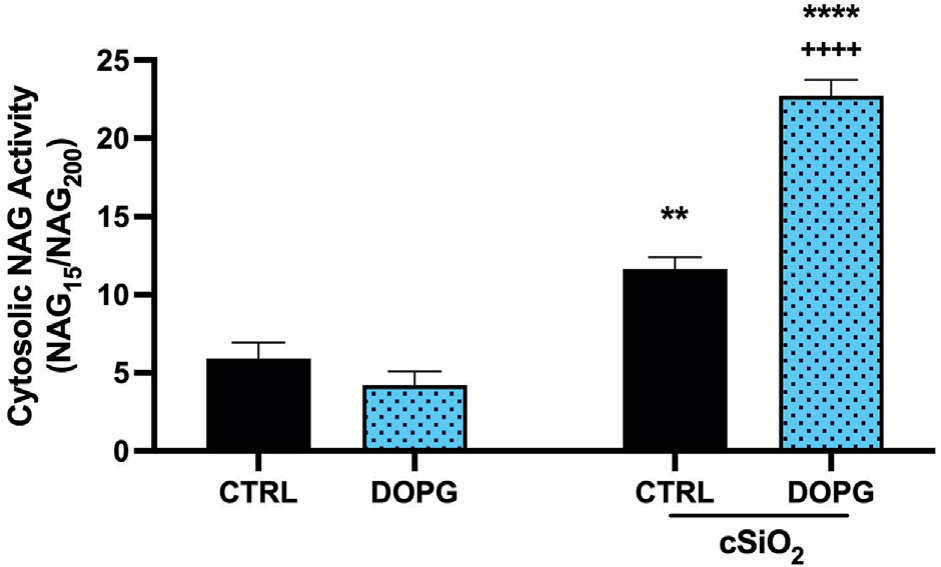
DOPG increases silica-induced LMP. BMdM treated with and without DOPG for 1 h before silica application for 24 h. BMdM extracted by digitonin (15 μg/mL) and cytosolic NAG activity measured with fluorescent NAG substrate. NAG values from 15 μg/mL digitonin extraction normalized to complete lysis by 200 μg/mL digitonin to indicate cytosolic NAG activity. Data expressed as *mean* ± *SEM*, *n* = 3. Analyzed by two-way ANOVA with Tukey's multiple comparisons test. **p < 0.01 and ****p < 0.0001 compared to control untreated cells. ^++++^ *p* < 0.0001 compared to silica-exposed cells.

The indicated change in susceptibility to digitonin titration upon treatment of DOPG supported the hypothesis that DOPG decreased lysosomal cholesterol consistent with previous reports (Ilnytska et al., 2021a; Ilnytska et al., 2021b). Increased lysosomal cholesterol has been demonstrated to be protective of LMP from a variety of causes (Appelqvist et al., 2011; Reiners et al., 2011; Biswas et al., 2014; Fucho et al., 2014). In this work, DOPG treatment of BMdM promoted cholesterol efflux, which in turn increased silica-induced cell death and IL-1β release. Therefore, we assessed the impact of decreased lysosomal cholesterol with DOPG treatment on LMP following silica exposure. BMdM were pre-treated with and without DOPG 1 h prior to silica application for 24 h. Cytosolic NAG activity was assessed using digitonin in a similar manner as described above. The digitonin dose was optimized by the previous titration to determine a working concentration (15 μg/mL) that selectively permeabilized the plasma membrane while leaving intracellular membranes intact and was compared to a complete lysis with 200 μg/mL for each condition. NAG activity was then expressed as NAG_15_/NAG_200_. This was done to account for cell death caused by silica, which could cause lower NAG levels in the extracts of silica treated BMdM. Silica exposure significantly increased LMP compared to untreated BMdM (Figure 5). DOPG alone did not impact NAG activity; however, DOPG pre-treatment before silica exposure resulted in a significant increase in NAG activity compared to silica-only BMdM (Figure 5). These results demonstrate that DOPG significantly increased silica-caused LMP.

### 3.6 Silica-induced membrane disruption in liposomes

**FIGURE 6:**
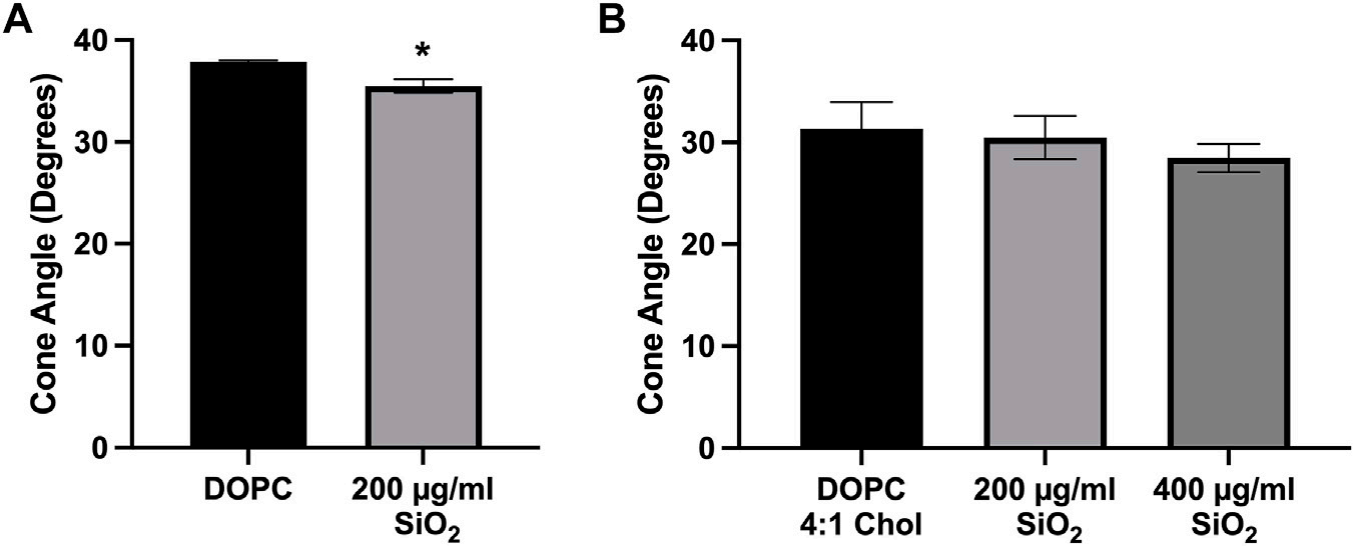
Time-resolved Anisotropy of Di-4ANEPPDHQ in Liposomes. **(A)** Pure DOPC liposomes treated with silica (200 μg/mL) for 2 h. **(B)** DOPC liposomes with cholesterol 4:1 M ratio incubated with silica (200 μg/mL and 400 μg/mL) for 2 h before anisotropy measurements were taken. Data expressed as *mean ± SEM*, *n* = 3 independent experiments. **(A)** Analyzed by one-way ANOVA with Dunnett’s *post hoc* test. **(B)** Analyzed with a two-tailed unpaired *t*-test. *p < 0.05.

We have previously demonstrated that crystalline silica can interact with and disrupt lipid membranes, which resulted in changes to lipid order in the membrane. This is likely through preferential interactions with phosphatidylcholine (DOPC) phospholipid headgroups (Pavan et al., 2020; Sydor et al., 2021; Pavan et al., 2022). Because of this our current work utilized 100-nm liposomes composed of DOPC with or without cholesterol that were incubated with crystalline silica for the purpose of detecting silica-induced changes to membrane order. Time-resolved fluorescence anisotropy was used to measure the movement of fluorescent membrane probe Di-4-ANEPPDHQ. The wobble-in-a-cone angle of Di-4-A-NEPPDHQ is reduced when the lipid order of the membrane is increased and prevents the movement of the probe within the membrane. Treatment with silica (200 μg/mL) caused a significant reduction of the cone angle compared to control DOPC liposomes (Figure 6A). In order to assess the effects of cholesterol on mitigating silica induced membrane disruption, DOPC liposomes were prepared with a 4:1 DOPC to cholesterol molar ratio. Addition of cholesterol to the liposomes prevented the decrease in cone angle with 200 μg/mL silica (Figure 6B). Addition of 400 μg/mL silica to the DOPC 4:1 Chol liposome also failed to significantly decrease the cone angle of Di-4-ANEPPDHQ (Figure 6B).

These results demonstrate that silica causes an increase in membrane lipid order as indicated by the reduced Di-4-ANEPPDHQ cone angle (Figure 6A) that is prevented by the addition of cholesterol to the DOPC liposome (Figure 6B).

## 4 Discussion

This study examined the mechanisms of silica-induced LMP and the role that lysosomal cholesterol content plays in mediating LMP. Lysosomal cholesterol content was increased with the cholesterol transport inhibitor U18666A. In order to promote a reduction in lysosomal cholesterol, BMdM were treated with DOPG liposomes. Phosphatidylglycerol is a precursor to the lysosomal lipid LBPA, which has been reported to stimulate cholesterol trafficking from the lysosome in NPC1 deficient cell models and directly interact with NPC2 (McCauliff et al., 2019; Ilnytska et al., 2021a; Ilnytska et al., 2021b). In the current work, the cells used were wild type BMdM from C57BL/6J mice, which have no deficiencies in their NPC1 and NPC2 proteins. The results in this study indicate that BMdM treatment with DOPG reduced lysosomal cholesterol content.

Macrophages, when exposed to crystalline silica, have been demonstrated *in vitro* and *in vivo* to phagocytose silica resulting in cell death, LMP, and IL-1β release (Gilberti et al., 2008; Biswas et al., 2017; Jessop et al., 2017; Ray et al., 2020). In the current work, pre-treatment of BMdM with U18666A reduced silica-induced cell death along with IL-1β release. This is proposed to be due to increased phagolysosomal cholesterol content that is attenuating silica-induced LMP. Conversely, pre-treatment of BMdM with DOPG liposomes produced an opposite effect to that of U18666A with significantly more cell death and IL-1β secretion than cells that had no pre-treatment prior to silica exposure. The critical role of cholesterol in regulating the outcomes was evaluated by applying U18666A or DOPG to the non-silica exposed cells which showed diverging effects on cholesterol efflux. U18666A reduced cholesterol efflux, which is attributed to a lack of cholesterol transport from the lysosome due to inhibited NPC1 activity (Lu et al., 2015). Treatment with DOPG produced an increase in supernatant cholesterol, which may be due to increased cholesterol trafficking from the lysosome (Ilnytska et al., 2021a; Ilnytska et al., 2021b).

To describe the effects of DOPG on the lysosome, we first measured the susceptibility of DOPG treated BMdM to permeabilization by digitonin. As the mechanism for digitonin to permeabilize the membrane is based upon membrane cholesterol content, this assay was suitable for examining how DOPG affected cholesterol content in both the plasma membrane and lysosomal membrane (Nishikawa et al., 1984; Sudji et al., 2015). We found that DOPG treatment had no effect on the plasma membrane cholesterol as demonstrated by the comparable LDH values of the digitonin titration between control and DOPG treated BMdM. However, the levels of NAG in the digitonin extracted cells were lower with DOPG treatment, indicating a reduction in lysosomal cholesterol. As decreased lysosomal cholesterol is proposed to cause an increase in silica-induced LMP, this same digitonin extraction technique was then utilized to quantify LMP. The comparison of NAG values for the working concentration of digitonin (15 μg/mL) that only permeabilized the plasma membrane was compared to the NAG values of complete cellular membrane lysis by 200 μg/mL digitonin. The fraction of cytosolically active NAG (NAG_15_/NAG_200_) demonstrated that silica caused a significant increase in LMP that was further exacerbated by DOPG treatment (Figure 5). Our results with lowered lysosomal cholesterol by DOPG make sense in the context of previous studies that showed U18666A-induced lysosomal cholesterol accumulation caused a decrease in susceptibility to LMP caused by various agents (Appelqvist et al., 2011; Reiners et al., 2011; Appelqvist et al., 2012). While different lysosomal sensitizers were used in these studies, the commonality remains that lysosomal cholesterol accumulation seemed to increase lysosomal membrane stability and prevent LMP.

In order to further investigate the effects of both U18666A and DOPG on lysosomes, filipin staining was used. After imaging the cells with confocal microscopy, an increase in filipin stained puncta was observed with U18666A treatment. This is likely due to a lack of lysosomal cholesterol trafficking, leading to increased filipin staining of lysosomes. When quantified, the number of puncta per cell was significantly increased with U18666A application. BMdM were also co-treated with U18666A and DOPG liposomes, which produced a significant increase in puncta per cell when compared to non-treated cells but a significant reduction in puncta compared to U18666A only treated cells. The filipin staining results indicates DOPG can reduce lysosomal cholesterol despite NPC1 being inhibited by U18666A. Treatment with only DOPG alone did not produce a change in filipin intensity and puncta when compared to control cells (data not shown) as control levels of filipin intensity and puncta per cell were already quite low.

Silica has been described to interact with lipid membranes and disrupt them, as measured by lipid order changes (Sydor et al., 2021; Pavan et al., 2022). Specifically, we have proposed that nearly free silanol groups on the surface of crystalline silica can interact with phosphatidylcholine headgroup lipids and cause a change in lipid order in membranes (Pavan et al., 2022). Liposomes were used as model membranes to examine how membrane cholesterol content impacts silica-induced membrane disruption. The wobble-in-a-cone angle of Di-4-ANEPPDHQ as measured by time-resolved fluorescence anisotropy was used as a gauge of lipid order within the liposome membrane. Liposomes produced with a mixture of DOPC and cholesterol at a 4:1 M ratio showed no significant change to lipid order after incubation with silica. However, DOPC liposomes with no cholesterol showed a significant reduction to the cone angle of Di-4-ANEPPDHQ. This indicates an interaction of the membrane with silica that increases lipid order and the ability of cholesterol to attenuate silica-induced membrane disruption. This interaction between particle and phospholipid head group is thought to restrict lipid motion and also has similarity to a local gelation that has been described for other particle-membrane interactions (Liu and Liu, 2020). While a seemingly large dose of silica was used in the current model membrane studies, it is important to consider the differences between liposomes and cells. Liposomes would interact with particles in a passive manner, while macrophages have active mechanisms to phagocytose the material, concentrating it in lysosomes. Therefore, the amount of material required to achieve and effect in each system would be expected to differ. Any affinity between Di-4-ANEPPDHQ and silica is not expected to cause a significant alteration to the wobble-in-a-cone angle. Changes to fluorescence anisotropy seem to be dictated more by phospholipid headgroups in the membrane, as Di-4-ANEPPDHQ in DOPS liposome membranes was not affected by silica treatment as described in a previous study (Pavan et al., 2022). While this study focused on one type of silica (⍺-quartz min-u-sil 5), other types of silica can share similar surface properties such as NFS. It may be the presence of these surface groups that dictates the bioactivity of different silica materials. Future work will need to utilize other types of materials aside from ⍺-quartz to assess the ability of lysosomal cholesterol to regulate LMP induced by other types of particles.

Lysosomes are critical regulators of cell homeostasis and play a role in modulating inflammatory cytokine release. Lysosomal dysfunction contributes to many inflammatory diseases including Alzheimer’s Disease, Parkinson’s Disease, Niemann Pick Disease, and silicosis (Vance and Karten, 2014; Hwang et al., 2019; Wie et al., 2021). In silicosis, lysosome dysfunction occurs when silica particles interact with specific phospholipid headgroups and change lysosomal membrane order as demonstrated with the liposome models used herein. Disrupting silica particle effects on lysosomal membranes by increasing cholesterol provides a therapeutic benefit in silica particle induced inflammation that is negated by DOPG treatment. This examination of lysosomal cholesterol provides insight into the mechanisms that contribute to particle induced inflammation and demonstrates that mechanistic understanding of lysosomal function in disease can provide therapeutic targets. Increasing targeted lysosomal cholesterol in diseases promoted by LMP could prove beneficial while decreased lysosomal cholesterol could prove detrimental.

## 5 Equations

The wobble-in-a-cone angle (*θ*) of Di-4-ANEPPDHQ was calculated by the equation below. The order parameter of the lipid membrane is represented by the value S. The initial anisotropy of Di-4-ANEPPDHQ is represented by *r*_*0*_, while the limiting anisotropy of the lipid membrane is defined by *r*_*∞*_. A wobble-in-a-cone angle can be generated by using both the initial and limiting anisotropy values and has been described (Kinosita Jr and Ikegami, 1982).

$$S^{2}=\frac{r_{\infty }}{r_{o}}={\left[\frac{1}{2}\cos\left(\theta \right)\left(1+\cos\left(\theta \right)\right)\right]}^{2}$$

## Data Availability

The raw data supporting the conclusion of this article will be made available by the authors, without undue reservation.
