# Supplementary material for: Cholesterol content regulates silica-induced lysosomal membrane permeability
Source: Front Toxicol. 2023 Feb 13;5:1112822. doi: 10.3389/ftox.2023.1112822 (PMC9969097; doi:10.3389/ftox.2023.1112822)
Supplement: Supplementary file 1 [file Table1.docx]

Supplementary Material

Cholesterol Content Regulates Silica-induced Lysosomal Membrane Permeability

Matthew J. Sydor*, Rebekah L. Kendall, Andrij Holian

*** Correspondence:** Corresponding Author: matthew.sydor@umontana.edu

# Supplemental Figures


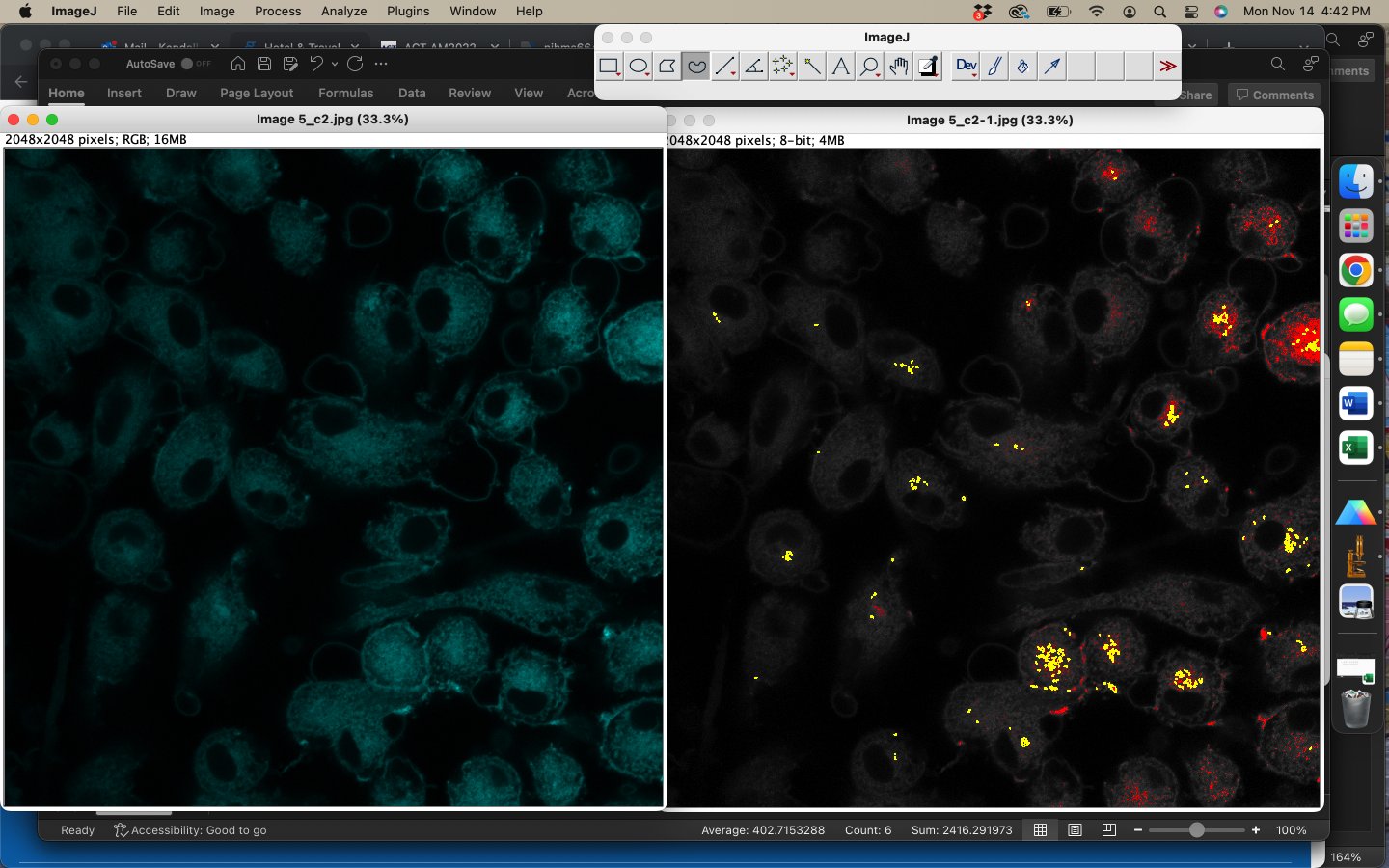
A.

B.


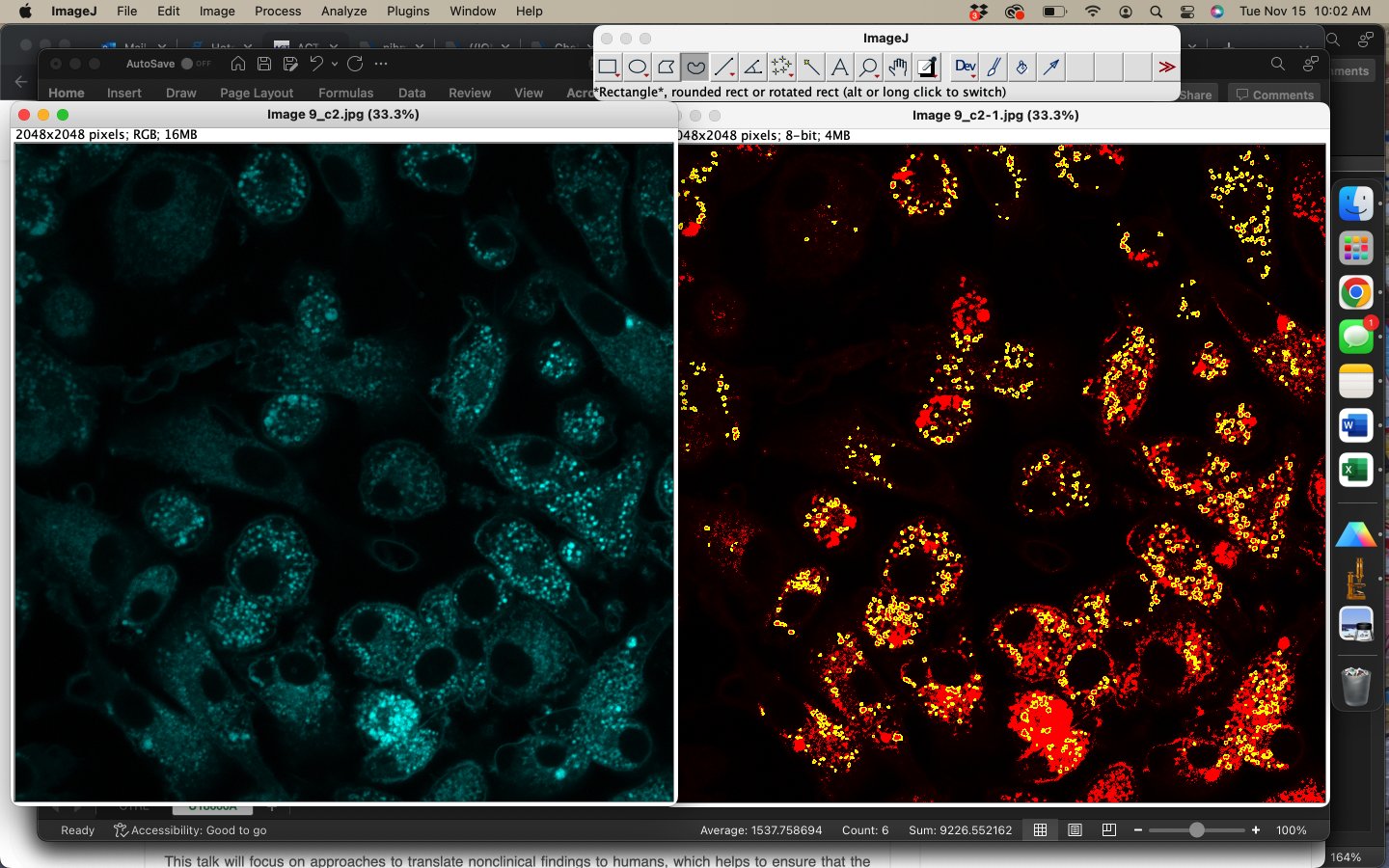


**Supplemental Figure 1. ImageJ quantification of puncta for control cells.** Puncta from filipin-stained images analyzed with ImageJ. Wand tracing tool used to select each cell, minimum intensity threshold set for cell, then Analyze Particle function utilized with a size threshold of 10-500 pixels^2^. Puncta as defined by intensity and size thresholds indicated by yellow coloring on above screenshot. A) Shows control BMdM. B) Shows U18666A-treated BMdM.

**
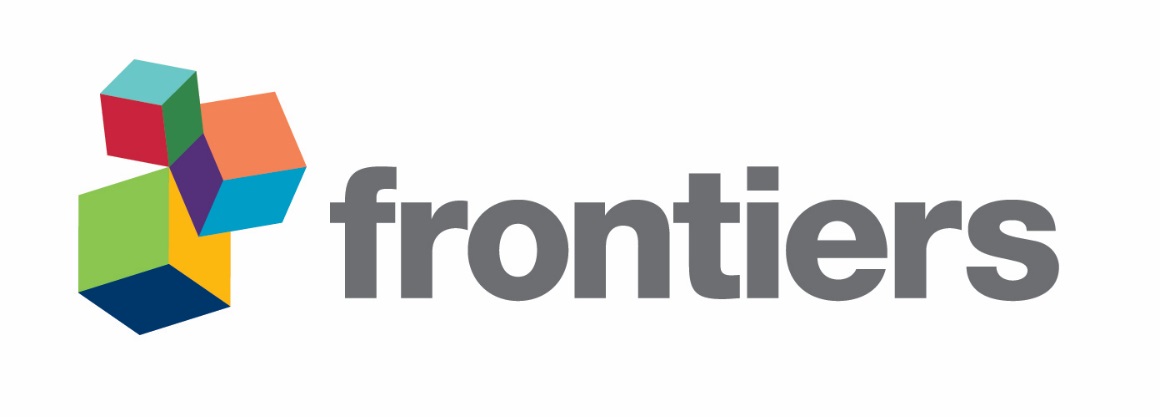
**

**
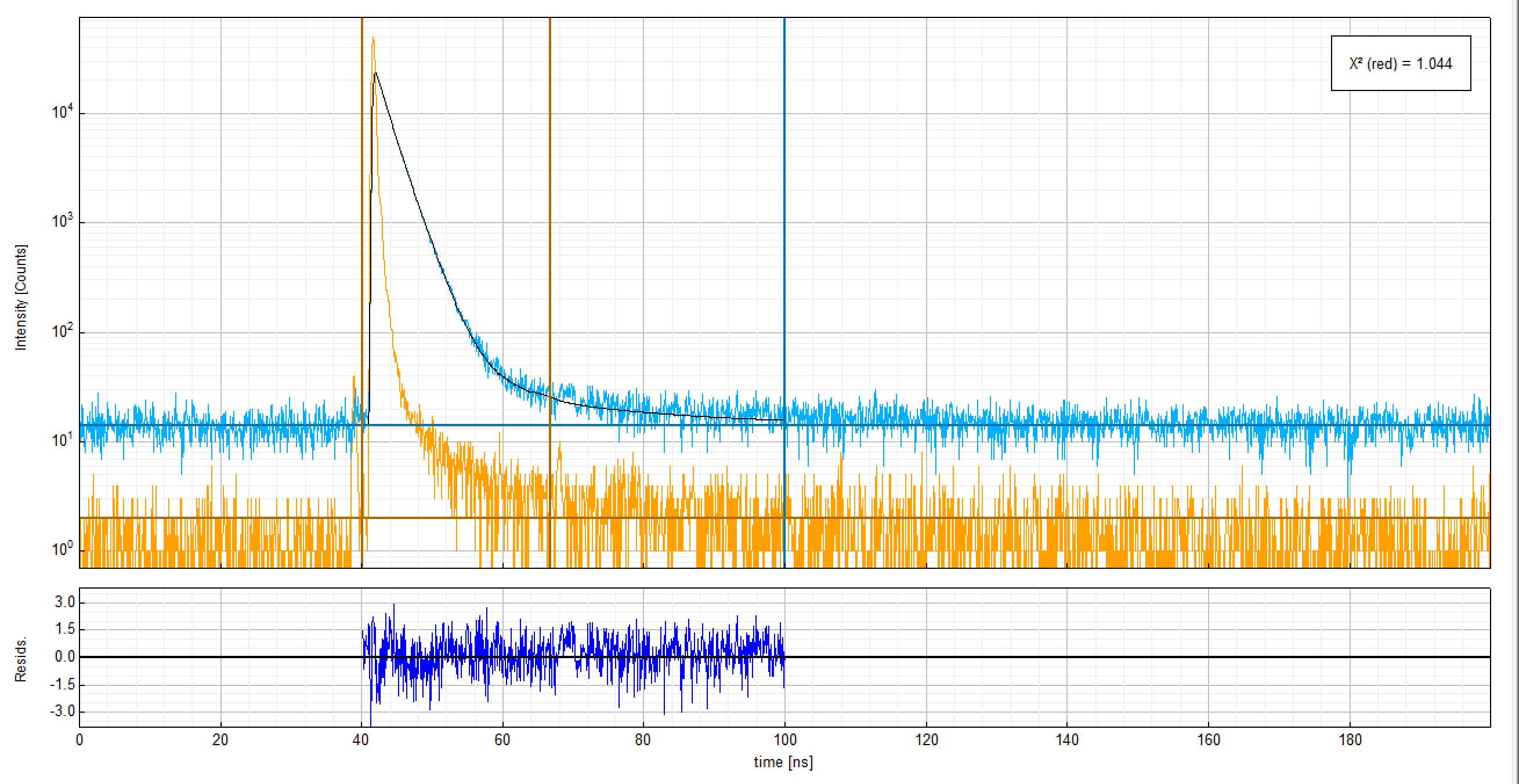
**

**
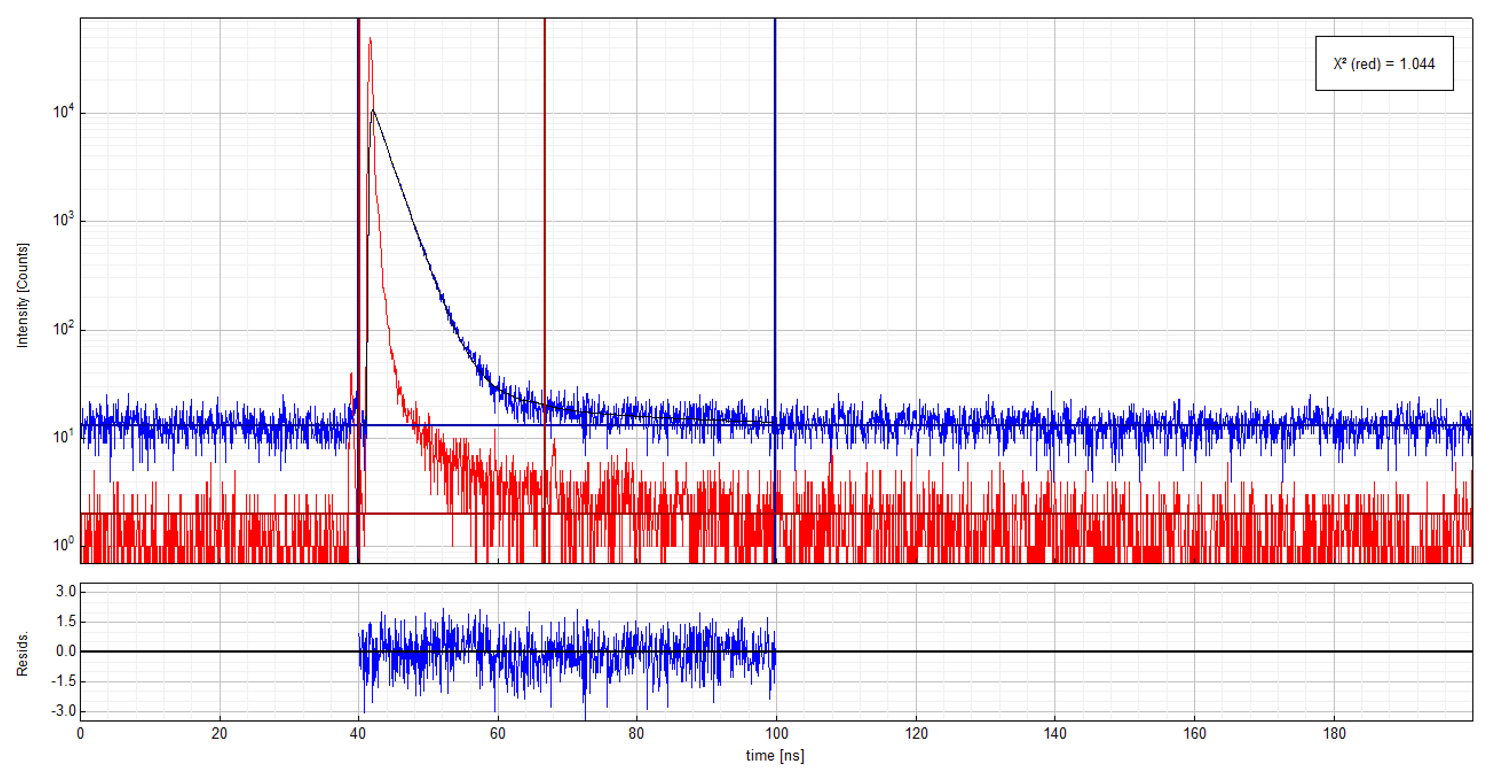
**

**Supplemental Figure 2.** **Fitting of Time-resolved Anisotropy Data.** The top image shows the fitted curve for vertically polarized emissions, while the bottom graph shows horizontally polarized emissions. These decays were fit with PicoQuant FluoFit software v4.6.6. The chi square value is shown in the top right corner.
